# Effects of combined jaw and cervicoscapular exercises on mouth opening and muscle properties in cervical extension type

**DOI:** 10.1038/s41598-025-03846-3

**Published:** 2025-05-30

**Authors:** Li-Jun Yu, Xin Yan, Tae-Ho Kim

**Affiliations:** 1 Department of Clinical Medicine, Suzhou Vocational Health College, No. 28 Ke Hua Road, Suzhou New District, Suzhou, 215009 China; 2 Department of Rehabilitation Science, The Graduate School, Daegu University, Gyeongsan-si, 38453 South Korea; 3 Department of Physical Therapy, College of Rehabilitation Science, Daegu University, Gyeongsan-si, 38453 South Korea

**Keywords:** Cervical extension type, Mouth opening range of motion, Temporomandibular joint, Health care, Health occupations

## Abstract

Prolonged smartphone use can lead to cervical posture deformities, with cervical extension type being a common condition characterized by increased cervical lordosis, forward head posture, and thoracic kyphosis. These changes may contribute to neck pain, restricted cervical range of motion (ROM), and increased muscle tone. Additionally, cervical extension type is linked to temporomandibular joint (TMJ) dysfunction, affecting mandibular movement and muscle activity. Given the biomechanical connection between the cervical spine and TMJ, addressing cervical dysfunction may benefit TMJ related conditions. This study compared the effects of jaw exercises combined with cervicoscapular exercises versus cervicoscapular exercises alone on mouth opening ROM, mastication muscle properties, and pressure pain threshold (PPT) in individuals with cervical extension type. Thirty-four subjects were randomly assigned to two groups: the experimental group (seventeen subjects) performed jaw exercises combined with cervicoscapular exercises, while the control group (seventeen subjects) performed only cervicoscapular exercises. After 4 weeks, significant improvements were observed in both groups in the mouth opening ROM, muscle properties, and PPT (*p* < 0.05). The experimental group showed significantly greater improvements in protrusive excursion, the masseter muscle tone, and the stiffness of the masseter and temporalis anterior muscles compared to the control group (*p* < 0.025). Both groups demonstrated significant increases in the PPT (*p* < 0.05). These findings suggest that incorporating jaw exercises into cervicoscapular training may provide additional benefits for individuals with cervical extension type, particularly those experiencing temporomandibular joint (TMJ) dysfunction. Further studies are needed to validate these results in a larger and more diverse population.

## Introduction

Prolonged smartphone use can contribute to cervical posture deformities and related issues. One of the most common conditions is the cervical extension type, which is marked by increased cervical lordosis, forward head posture, and thoracic kyphosis. These biomechanical alterations may result in neck pain, limited range of motion (ROM), and increased cervical muscle tone^1^.

Prolonged head flexion can lead to forward head posture, impair cervical ROM, and affect balance and head control, leading to increased mechanical load and dysfunction^2^. Cervical extension type causes sustained cervical spine loading, which leads to changes in the length–tension relationship of the anterior and posterior muscles of the neck, capsuloligamentous structures, and mechanoreceptors, in turn, negatively influencing muscle spindle activity, considered important for head position sense^3^.

The literature states that cervical extension type combined with comorbid acute and chronic cervical pain among subjects who sit for a long time contributes to changes in the myofascial tone and tensegrity as well as aggravated pressure sensitivity in affected muscles^4^.

In some studies, it has been suggested that excessive cervical extension type affects the head’s center of gravity position and the position of the mandible in the temporomandibular joint (TMJ), gradually leading to dysfunction. According to those authors, excessive cervical extension type disrupts the mechanics of the cervical spine and may affect deep muscle tension^5^. TMJ dysfunction affects approximately 34% of the global population. Moreover, disorders of the cervical spine and TMJ are often associated with nocturnal bruxism and teeth grinding during the night. The comorbidity of bruxism and TMD is estimated to have a prevalence of around 17%^6^. TMJ dysfunction is characterized by limited mandibular movements and can involve joint sounds, pain, and altered sensitivity and muscle activity^7^. Assessing mouth opening is an important part of the basic examination of subjects completed by clinicians treating head and neck disorders^8^.

The upper cervical spine, also known as the craniovertebral joint, plays a key role in sensing movement and position. Changes in head posture can influence mandibular position due to muscular and joint interconnections^9^. Mandibular movement works in synchrony with head movements as well as masticatory and cervical muscle activation. The relationship between the craniocervical region and the dynamics of the TMJ has been supported in previous studies^10^. Myofascial trigger points in the upper trapezius can affect masticatory muscle activity, leading to asymmetry and dysfunction. This highlights a functional link between the cervical spine and temporomandibular joint, suggesting cervical issues may contribute to TMDs and tension-type headaches^11^. Almoznino et al. (2020) stated that “the study demonstrated a significant association between temporomandibular disorders (TMD) and cervical muscle tenderness, particularly in patients with myogenous TMD. Cervical tenderness was statistically correlated with the severity of TMD symptoms, including masticatory muscle tenderness, pain intensity, headache, and widespread pain. Furthermore, painful mouth opening, history of whiplash, and female gender were significantly associated with cervical tenderness. These findings suggest a close functional relationship between the temporomandibular joint and cervical spine musculature, highlighting the importance of considering cervical involvement in the assessment and management of TMD patients”^12^.

For cervical dysfunction, various posture-improving interventions, such as stretching, strengthening exercises, and posture re-education techniques, have been shown to effectively enhance cervical function^13–16^. Additionally, combined cervical and shoulder exercises, including stabilization training, have proven effective in improving musculoskeletal health^17–20^.

For TMJ dysfunction, therapeutic approaches such as splint therapy, jaw exercises, and neuromuscular control training have also demonstrated efficacy^21–24^.

To the best of our knowledge, few studies have specifically examined the combined effects of jaw and cervicoscapular exercises in individuals with cervical dysfunction, despite the well-established interrelationship between cervical spine and TMJ function. While existing research has addressed cervical or TMJ dysfunction independently, limited evidence is available regarding their integrated management, particularly in individuals with cervical extension-type posture.

Therefore, this study aims to investigate the effects of a combined intervention involving cervicoscapular complex exercises and jaw exercises on the mechanical properties of the cervical and masticatory muscles in individuals with cervical extension-type posture. Specifically, we assess changes in muscle elasticity, tone, and stiffness in the temporalis anterior and masseter muscles. We hypothesize that participants undergoing the combined intervention will show greater improvements in these muscle properties—characterized by decreased stiffness and tone and increased elasticity—compared to those performing cervicoscapular exercises alone. Additionally, we hypothesize that the combined intervention will significantly improve mouth opening range, reflecting enhanced functional mobility of the masticatory system.

## Methods

### Participants

This study was conducted with individuals with cervical extension type who were attending Daegu University in Gyeongsan, South Korea. Prior to participation, all subjects read and signed a consent form approved by the Institutional Review Board of Daegu University (IRB Number: 1040621-202301-HR-025). All participants provided informed consent to participate in the study, and the data were handled according to the ethical standards of the Declaration of Helsinki. Written informed consent was obtained from all participants before their enrollment in the study.

The inclusion criteria employed were as follows: (1) A craniovertebral angle (CVA) of ≤ 53°, which has been identified in previous studies as indicative of forward head posture, particularly reflecting a cervical extension-type posture^1^; (2) A cranial rotation angle > 143°, representing excessive extension and rotation of the head and neck that is characteristic of this posture type^25^. These angular thresholds were selected based on established research to ensure the precise identification of individuals with cervical extension-type forward head posture. The use of these criteria helped enhance the specificity and clinical relevance of the sample population. Exclusion criteria included individuals with a history of traumatic neck injury, inflammatory joint disease, cervical spine infection, severe osteoporosis, cervical disc protrusion, nerve root compression, cervical fracture or dislocation, prior cervical surgery, severe migraine, vestibular disorders, or vertebrobasilar insufficiency^3^.

The required sample size was calculated using G*Power version 3.1, based on a repeated measures ANOVA with a within–between interaction. A medium effect size (f = 0.25) was adopted in accordance with Cohen’s recommendations, which are commonly used in behavioral and clinical studies when prior effect size estimates are limited. The significance level was set at α = 0.05, and the statistical power was set at 0.90 to reduce the risk of Type II error and ensure a high probability of detecting a clinically meaningful effect. The analysis indicated that a minimum of 30 participants was required^26^.

### Study procedure

The experiment was conducted from March 1 to May 1, 2023, with 39 participants initially screened. Five were excluded due to a craniovertebral angle greater than 53°, leaving thirty-four participants with cervical extension type. These were randomly assigned to two groups: the experimental group (*n* = 17) received cervicoscapular complex exercises combined with jaw exercises, while the control group (*n* = 17) performed only cervicoscapular exercises. Both groups followed the same four-week exercise regimen, with sessions three times per week. Random assignment of participants was conducted by an independent administrator who had no involvement in the experimental procedures. To ensure allocation concealment and an equal probability of group assignment, each participant drew one of two differently colored ping pong balls (yellow or white) from a sealed opaque container, corresponding to the experimental or control group. This simple randomization method guaranteed a 1:1 allocation ratio while maintaining transparency and reducing selection bias.

All assessments were performed by the same trained examiners before and after the intervention. To reduce detection bias, the assessors remained blinded to group allocation during the post-intervention evaluations. Group information was only disclosed to them after all outcome measurements were completed.

Pre- and post-intervention measurements included mouth opening range, muscle properties (elasticity, stiffness, and tone), and pressure pain threshold (PPT). All assessments were conducted by the same examiner at the end of the study.

### Measurements

#### Measurement of the mouth opening ROM

The range of mouth opening assessment was performed under two conditions: comfortable mouth opening and maximum mouth opening^27^. For the measurement of maximum and comfortable mouth opening, the subject was positioned in an upright chair with a backrest, facing forward. To measure the maximum mouth opening, the ex-aminer instructed the subject to “open your mouth as wide as you can.” During this instruction, the distance between the upper and lower teeth was measured^28^. To measure the comfortable mouth movement, the examiner instructed the subject to “slowly open your mouth until I tell you to stop.” While the subject followed this instruction, the examiner palpated both mandibular condyles using both hands. The examiner then indicated the subject to stop before the condyles were translated forward, and the distance between the upper and lower teeth was measured^28^. The distance between the first right incisor of the maxilla and mandibular^29^was measured with an electronic digital caliper^30^.

To assess the maximal laterotrusive movement to the left and right, the posterior teeth were brought into the maximal intercuspal position. A vertical line was drawn using a pencil between the mesiallabial surfaces of the maxillary central incisors, representing the maxillary midline. This line was extended onto the labial surface of the opposing mandibular antagonistic incisor, taking into account any possible discrepancies between the maxillary and mandibular midlines. The subject was then instructed to move their mandible as wide as possible to the left and to the right. The lateral excursion measurements were recorded between the two lines, with measurements taken in 1 mm incre-ments^30^.

The starting position for measurement was the physiological rest position, from which the subject moved the mandible anteriorly without teeth contact. The distance between the incisal edge of the maxillary central incisor and the incisor edge of the mandibular incisor was measured in the maximum protruded position^29^ in 1 mm steps^30^.

#### Measurement of the mechanical properties

The muscles that were measured were the temporal muscle and the masseter muscle on both sides. To assess the dominant and non-dominant sides of the facial masticatory muscles, participants were instructed to chew gum. The side that was more comfortable for the subjects to feel was the dominant side and the side that was selected. Before the study, we marked the skin on the highest point of the muscle belly of each measured muscle and took measurements by placing the myometer vertically on this mark^31^.

#### Measurement of the muscle PPT

PPT of the anterior temporalis and masseter muscles was measured using a digital pressure algometer (Mecmesin Compact Force Gauge, 500 N, UK). For the anterior temporalis muscle, the measurement point was located approximately 2 cm posterior to its anterior border along a line from the superior orbital margin to the top of the auricle. For the masseter muscle, the measurement site was the thickest portion of the muscle belly, identified during voluntary clenching^27^. The measurement sites were marked with a skin-safe marker prior to testing. The algometer was calibrated according to the manufacturer’s guidelines. It was factory-calibrated using standardized weights traceable to the National Institute of Standards and Technology (NIST), and a zero-calibration check was automatically performed at each startup to ensure measurement accuracy and minimize baseline drift. To ensure measurement repeatability, each site was tested three times, with a 30-second rest interval between measurements. A 3-minute rest was provided between measurements of different muscle sites to prevent sensitization. Pressure was applied perpendicularly to the muscle surface at a constant rate of 0.5 kgf/cm² per second, which was standardized using a metronome set at 60 beats per minute. Participants were instructed to say “stop” at the moment they first perceived pain. The corresponding pressure value was automatically recorded by the device and expressed in kgf/cm². The average of the three trials was used for analysis^1^.

### Intervention

#### Jaw exercises

Jaw exercises include suboccipital muscle stretching, masseter stretching, digastric facilitation exercises, cross-fingered exercise, infrahyoid muscle strengthening, and suprahyoid muscle stretching. The motion method used in this study was compiled and modified from the motion methods of various studies^32–34^.

Each exercise should be performed for a duration of 30 s, and it is recommended that each exercise is repeated 5 times. This constitutes two rounds of the exercises, taking approximately half an hour to complete^33^ (Table 1).

**Table 1:** Jaw exercises.

To effectively stretch the suboccipital muscles, place one hand on the back of your head and gently apply a downward and forward pressure. Slowly tilt your head forward, bringing your chin toward your chest while maintaining steady pressure.	Start with your mouth closed and relax your jaw as much as possible. Then, gently and slowly open your mouth as wide as you comfortably can, maintaining a smooth and controlled movement.
Suboccipital muscle stretching	Masseter stretching
The subjects were instructed to open and close their mouths while pressing their tongues against the palate. Additionally, they placed their hands on the temporomandibular joint to enhance awareness of movement. They were guided to open their mouths slowly and in a controlled manner.	The thumb and index finger were used to assist in gently and gradually opening the mouth in a controlled manner.
Digastric facilitation exercises (goldfish exercise)	Cross-fingered exercise
Mouth opening was initiated from a closed position, with manual pressure applied to the lower jaw to assist the movement.	Using the fingers, gently position them beneath the jaw near the first hyoid bone. Pinch the muscles in this area with the first and second fingers while ensuring contact with the bone. As the muscles are pinched, tilt the head upward. Discomfort during this movement may indicate the need for stretching. Next, identify the muscles just above the hyoid bone and, while opening the mouth, simulate the motion of yawning.
Infrahyoid muscle strengthen	Suprahyoid muscle stretching

#### Cervicoscapular complex exercises

Cervicoscapular complex exercises were mainly aimed at improving the cervical, shoulder, and thoracic muscles. They mainly consisted of cervical exercises and shoulder exercises. The motion method used in this study was compiled and modified from the motion methods of various studies^35–40^.

The cervical exercises included chin tucks, nuchal ligament stretching, sagittal rotation (sitting/quadruped), deep cervical flexor strengthening, and cervical rotation (Table 2). The shoulder exercises included upper trapezius stretching, sternocleidomastoid muscle stretching, levator scapular stretching, the wall sliding exercise, and thoracic extension exercises^41^ (Table 3).

In the control group, each session consisted of 3 sets of 10 repetitions, with a 10-second hold and a 2-minute rest between sets, lasting approximately one hour per session. These sessions were completed over 4 weeks. In the experimental group, each session also included 3 sets of 10 repetitions, but with a 5-second hold and the same 2-minute rest between sets. The total session duration for the experimental group was around 30 min, also following the 4-week protocol^38^.

**Table 2:** Cervical exercises completed as part of cervicoscapular complex exercises.

Cervical	Exercises
Chin tucks	Subjects were seated in a chair with their feet flat on the floor and shoulders relaxed. While maintaining a forward gaze, they gently retracted their chin in a straight-back motion, ensuring a small and controlled movement.
Nuchal ligament stretching	In the chin-in position, one hand stabilizes the chin while the other hand is placed at the back of the head to apply a gentle downward and forward force.
Sagittal rotation (sitting)	In the seated position, the superficial neck muscles are relaxed to reduce activity, allowing the deep neck flexors and deep cervical muscles to be engaged during sagittal plane rotation training. Instruct the subject to “roll your head in place, like a ball,” ensuring smooth and controlled movement.
Sagittal rotation (quadruped)	In the quadrupedal posture, flexion and extension of the head nod are first trained, and the sagittal plane of the neck extension and flexion is performed. Slowly perform the forward and backward sagittal plane rotation so that it is caused by the deep neck muscles.
Deep cervical flexor strengthening	Subjects were positioned in a supine lying posture, with the air unit of the pressure biofeedback device placed at the posterior aspect of the cervical spine, just below the occiput. The exercise targets the deep flexor muscles of the upper cervical region, focusing on these muscles rather than the superficial flexors, which primarily flex the neck without engaging the head.
Cervical rotation	Leaning the arm against the wall helps reduce the weight of the arm and promotes relaxation of the upper ipsilateral trapezius muscle. The subject is instructed to move slowly, allowing for controlled axial rotation. Sophisticated exercises are performed to prevent excessive neck stretching or flexion, ensuring a smooth and gradual movement.

**Table 3:** Shoulder exercises completed as part of cervicoscapular complex exercises.

Shoulder	Exercises
Upper trapezius muscle stretching	Sit upright and slowly tilt your head to the side, bringing your right ear toward your right shoulder. Keep your head straight, without turning or deviating, and maintain a forward gaze.
Sternocleidomastoid muscle stretching	Rotate your neck toward the opposite side you wish to stretch, then tilt your head away from that side while extending your head backward. Finally, gently push your jaw forward to increase the tension on the muscle.
Levator scapulae stretching	For this stretch, the subject turns their head halfway and looks down toward their armpit. To increase the stretch, maintain the same position, keep looking down, and gently hold the head while applying slight pressure by pulling gently.
Wall sliding exercise	In the wall slide exercise, the lower arms glide upward against the wall with the shoulder joints at 90° and the elbows flexed at 90°, while the trunk remains fixed in a standing position. This movement aims to strengthen the serratus anterior muscle.
Thoracic extension exercise	To use a foam roller to increase range of motion, place it in the lower part of the back and support the neck area with both hands, ensuring the neck is not pulled forward. Begin the exercise by moving along the spine. It is important to maintain the distance between the xiphoid process and the symphysis pubis by contracting the abdominal muscles, as the movement should be focused in the back while minimizing movement in the waist area.

### Statistical analysis

Data were analyzed using IBM SPSS Statistics version 26.0 (IBM Corp., Armonk, NY, USA). Descriptive statistics (mean ± standard deviation) were used to summarize participant characteristics. The Shapiro–Wilk test was used to assess normality. Independent t-tests were conducted to compare baseline characteristics between groups.

A two-way repeated measures ANOVA was performed to analyze the main effects of time, group, and their interaction. Post hoc comparisons were conducted using the Bonferroni correction, with the significance level adjusted to α < 0.025.

Within-group changes before and after the intervention were analyzed using paired t-tests. Effect sizes were calculated using partial eta squared (η²), with thresholds of 0.01 (small), 0.06 (medium), and 0.14 (large). All analyses were performed with a significance level set at *p* < 0.05. Analyses were conducted by a blinded researcher not involved in the intervention.

## Results

A total of 34 subjects were enrolled in this study and were assigned to either the EG or the CG. No statistically significant differences were observed between the general characteristics of the two groups (Table 4).

**Table 4:** General characteristics of subjects (*n* = 34).

Variable	EG	CG	t(*p*)
Age(year)	24.06 ± 2.30	23.82 ± 3.63	0.226(0.823)
Sex(female/male)	10/7	8/9	−0.671(0.507)
Height(cm)	167.18 ± 7.27	172.41 ± 10.16	−1.728(0.094)
Weight(kg)	62.90 ± 10.62	72.15 ± 24.77	−1.416(0.171)
BMI(kg/m^2^)	22.38 ± 2.36	23.77 ± 6.01	−0.892(0.383)
CVA(°)	46.64 ± 6.93	41.66 ± 9.88	1.702(0.100)
CRA(°)	153.88 ± 8.38	157.04 ± 8.13	−1.116(0.273)

Values are expressed as mean ± SD. CVA: Craniovertebral angle; CRA: Cranial rotation angle; BMI: Body mass index; EG: Cervicoscapular complex exercises with jaw exercises; CG: Cervicoscapular complex exercises; *p < 0.05.

All groups showed significant differences in the mouth ROM, the mechanical properties of the MM and TA muscles, and PPT (*p* < 0.001) (Tables 5, 6 and 7).

Mouth opening ROM showed significant improvements in the following measurements: CMO (F = 86.168, *p* < 0.001, η² = 0.729), MMO (F = 74.631, *p* < 0.001, η² = 0.700), LLE (F = 55.939, *p* < 0.001, η² = 0.636), RLE (F = 28.469, *p* < 0.001, η² = 0.471), and PE (F = 41.651, *p* < 0.001, η² = 0.566) Additionally, a significant group-by-time interaction was observed for PE (F = 10.660, *p* = 0.003, η² = 0.250) (Table 5).

There were also significant time interactions for muscle tone, stiffness, elasticity, and PPT in both the MM and TA muscles. In the MM, muscle tone significantly decreased (F = 71.661, *p* < 0.001, η² = 0.691), stiffness decreased (F = 87.796, *p* < 0.001, η² = 0.733), and elasticity improved (F = 85.409, *p* < 0.001, η² = 0.727). PPT also significantly increased (F = 32.347, *p* < 0.001, η² = 0.503). Similarly, in the TA muscle, tone significantly decreased (F = 44.574, *p* < 0.001, η² = 0.582), stiffness decrease (F = 43.130, *p* < 0.001, η² = 0.574), elasticity improved (F = 42.197, *p* < 0.001, η² = 0.569), and PPT increased (F = 37.713, *p* < 0.001, η² = 0.541). Significant group-by-time interactions were also observed. In the MM, significant interactions were found for muscle tone (F = 10.487, *p* = 0.003, η² = 0.247) and stiffness (F = 7.102, *p* = 0.012, η² = 0.182). In the TA, significant interactions were noted for stiffness (F = 6.145, *p* = 0.019, η² = 0.161) and elasticity (F = 5.680, *p* = 0.007, η² = 0.172) (Tables 6 and 7).

**Table 5:** Change in mouth ROM in the two groups (unit: ◦) (*n* = 34).

		Pre	Post	t(*p*)	Time F(*p*)	Group × Time F (*p*)
CMO	EG	24.87 ± 6.86	33.87 ± 5.66	−9.987(< 0.001*)	86.168 (< 0.001*)	2.149 (0.152)
	CG	22.09 ± 7.20	28.64 ± 5.31	−4.637(< 0.001*)		
	η^2^				0.729	0.063
MMO	EG	40.71 ± 6.30	48.07 ± 4.92	−6.983(< 0.001*)	74.631 (< 0.001*)	0.814 (0.374)
	CG	36.64 ± 8.84	42.61 ± 7.58	−5.297(< 0.001*)		
	η^2^				0.700	0.025
LLE	EG	6.94 ± 0.90	9.89 ± 2.54	−4.985(< 0.001*)	55.939 (< 0.001*)	0.002 (0.963)
	CG	6.66 ± 1.30	9.57 ± 1.92	−5.666(< 0.001*)		
	η^2^				0.636	0.000
RLE	EG	7.50 ± 2.15	9.40 ± 2.36	−3.957(0.001*)	28.469 (< 0.001*)	0.308 (0.583)
	CG	7.79 ± 1.57	9.33 ± 1.76	−3.580(0.003*)		
	η^2^				0.471	0.010
PE	EG	5.42 ± 1.97	7.82 ± 1.45	−6.002(< 0.001*)	41.651 (< 0.001*)	10.660 (0.003)*
	CG	5.43 ± 1.54	6.22 ± 1.53	−2.716(0.015*)		
	η^2^				0.566	0.250

Values are expressed as mean ± SD. EG: Cervicoscapular complex exercises with jaw exercises; CG: Cervicoscapular complex exercises; CMO: Comfortable mouth opening; MMO: Maximum mouth opening; LLE: Left lateral excursions; RLE: Right lateral excursions; PE: Protrusion excursions; η^2^: effect size; *p < 0.05.

**Table 6:** Change in muscle mechanical properties in the two groups (unit: F: HZ S: N/m) (*n* = 34).

			Pre	Post	t(*p*)	Time F(*p*)	Group × Time F(*p*)
MM	F	EG	16.26 ± 1.58	14.44 ± 1.63	6.414(< 0.001*)	71.661 (< 0.001*)	10.487 (0.003*)
		CG	16.13 ± 1.98	15.32 ± 2.06	6.384(< 0.001*)		
		η^2^				0.691	0.247
	S	EG	341.00 ± 67.89	303.41 ± 64.28	7.538(< 0.001*)	87.796 (< 0.001*)	7.102 (0.012*)
		CG	323.41 ± 70.61	302.47 ± 65.04	5.567(< 0.001*)		
		η^2^				0.733	0.182
	D	EG	1.73 ± 0.25	1.88 ± 0.25	−7.900(< 0.001*)	85.409 (< 0.001*)	3.742 (0.062)
		CG	1.58 ± 0.25	1.68 ± 0.30	−5.169(< 0.001*)		
		η^2^				0.727	0.105
TA	F	EG	37.06 ± 5.15	34.48 ± 4.54	5.721(< 0.001*)	44.574 (< 0.001*)	0.973 (0.331)
		CG	36.05 ± 7.87	34.13 ± 7.43	3.831(0.001*)		
		η^2^				0.582	0.029
	S	EG	818.53 ± 198.89	657.53 ± 84.82	5.269(< 0.001*)	43.130 (< 0.001*)	6.145 (0.019*)
		CG	899.06 ± 372.85	826.29 ± 314.79	3.986(0.001*)		
		η^2^				0.574	0.161
	D	EG	1.59 ± 0.13	1.85 ± 0.23	−4.853(< 0.001*)	42.197 (< 0.001*)	1.805 (0.189)
		CG	1.68 ± 0.19	1.84 ± 0.20	−4.370(< 0.001*)		
		η^2^				0.569	0.053

Values are expressed as mean ± SD. EG: Cervicoscapular complex exercises with jaw exercises; CG: Cervicoscapular complex exercises; MM: Masseter muscle; TA: Temporal anterior; F: muscle tone; S: stiffness; D: elasticity; η2: effect size; *p < 0.05.

**Table 7:** Change in muscle PPT in the two groups (unit: lb/cm^2^) (*n* = 34).

		Pre	Post	t(*p*)	Time F(*p*)	Group × Time F(*p*)
MM	EG	3.34 ± 0.99	4.59 ± 1.49	−3.951(0.001*)	32.347 (< 0.001*)	0.879 (0.356)
	CG	3.57 ± 1.49	4.56 ± 2.01	−4.358(< 0.001*)		
	η^2^				0.503	0.027
TA	EG	3.98 ± 1.58	4.91 ± 1.96	−3.876(0.001*)	37.713 (< 0.001*)	0.232 (0.633)
	CG	3.94 ± 1.35	4.73 ± 1.69	−5.443(< 0.001*)		
	η^2^				0.541	0.007

Values are expressed as mean ± SD. EG: Cervicoscapular complex exercises with jaw exercises; CG: Cervicoscapular complex exercises; MM: Masseter muscle; TA: Temporal anterior; *p < 0.05.

## Discussion

The results of this study show that combined neck, shoulder stability training, and jaw exercises synergistically improve temporomandibular joint (TMJ) function, suggesting a close neuromuscular connection between the cervical, shoulder, and mandibular regions. This is consistent with previous research. Existing literature has pointed out that the cervical and masticatory muscle systems influence each other through the trigeminocervical nucleus, and improvements in cervical and shoulder muscle stability can indirectly alleviate compensatory overactivity of the masticatory muscles, thus improving jaw opening range, reducing muscle tone, and enhancing the pressure pain threshold^42^. Changes in muscle properties such as elasticity, tone, and stiffness are closely related to functional recovery^42^.

The average maximum interincisal opening and lateral and protrusive movement have been reported as 40–58 mm and 8–10 mm, respectively^44^. The normal range of movement of the temporomandibular joint is 4 ~ 5 cm, and if this is less than 3.5 cm, it is defined as TMJ dysfunction^23^. The present study showed significant improvements in mouth opening for both groups. Previous research has highlighted the effectiveness of exercises targeting the deep cervical flexors and exercises stretching the semispinalis ca-pi-tis, splenius capitis, sternocleidomastoid, and upper trapezius in increasing the jaw muscle flexibility and enhancing the mouth opening range of movement^43^. In a previous study, deep cervical flexor muscle strengthening was effectively improved, enhancing the mouth opening ROM^43^. Nociceptive impulses from the upper cervical spine cause reflex contractions in masticatory muscles, which can contribute to the development of TMJ dysfunction. Thus, exercises for the upper cervical region appear to reduce muscular reflex contractions and allow for muscle relaxation, especially in the masseter muscles, and may consequently increase the mouth opening ROM^43^. Posture training has been shown to improve TMJ dysfunction by stretching shortened muscles and strengthening weakened ones due to poor posture. It has also helped individuals develop an awareness of proper head, neck, and jaw alignment, addressing postural imbalances and alleviating the related symptoms of temporomandibular dysfunction^44^, consistent with the results of this study.

In the between-group comparison, the experimental group showed a significant improvement in the protrusion excursions compared to the control group. Mastication involves the coordinated activation of the jaw, tongue, and facial muscles. Jaw opening is driven by the anterior belly of the digastric and mylohyoid muscles, while protrusion re-quires lateral pterygoid, anterior temporalis, and superficial masseter muscle activation. Jaw retrusion is controlled by the posterior temporalis fibers^45^. In these muscles, the masseter is primarily responsible for the elevation and some protraction of the mandible^46,47^. Deep to the temporalis and masseter, the medial pterygoid further assists with mandibular elevation and protrusion via its attachments to the lateral pterygoid plate and medial surface of the ramus of the mandible^47^. The lateral pterygoid muscle is responsible for depressive, protrusive, and lateral excursive movements of the jaw^48^. In the experimental group, the cross-fingered exercises and masseter muscle stretching helped depress the mandible, while stretching the masseter and lateral pterygoid muscles relieved tension. The stretching was performed with active jaw opening and closing, applying pressure to the muscle fibers. This technique was also applied to the temporal and masseter muscles^49^. As a result, the relaxation of tonic muscles contributed to an in-crease in the jaw opening range^50^.

Muscle properties, such as elasticity, tone, and stiffness, are important components that can affect joint control and stability. In addition, muscle mechanical properties could affect the stretch–shortening cycle and have a potential effect on rapid force production during functional or dynamic movements^51^. In the intra-group comparison of the muscle elasticity of the masseter muscle and temporal anterior muscle, both groups showed a significant increase. Both groups of exercises that were performed targeted the stretching of the shortened muscles. Previous studies have shown that stretching can improve stiffness and increase elasticity^52^. Stretching stimulates the nervous system, al-lowing for better muscle activation and coordination, which can contribute to decreased stiffness, and a reduction in the stiffness of these muscles may allow for greater compliance with muscle contraction and therefore improve movement fluidity^53^. Regular stretching exercises and flexibility training can lead to an increase in muscle elasticity. When consistently stretching muscles, the muscle fibers and surrounding connective tis-sue adapt to the increased range of motion by becoming more elastic^54,55^.

The results on muscle tone in this study showed significant decreases in both groups before and after the intervention. In this study, the stretching exercises targeting the nuchal ligament, upper trapezius, sternocleidomastoid, and levator scapulae muscles proved effective in increasing the muscle length, range of motion, and reducing muscle tone in individuals with cervical extension type^56^, similar to previous findings. Additionally, both groups performed the thoracic extension exercises using a foam roller, which, through slow movements and pressure, helps relax the fascia and reduce muscle tone. Continuous, slow pressure on tissues stimulates mechanoreceptors, sending signals to the central and autonomic nervous systems to alleviate muscle tone^57^. The between-group comparisons revealed a greater reduction in the masseter muscle tone in the experimental group compared to that in the control group. All of the participants in the experimental group performed masseter and suboccipital muscle stretching exercises. Previous studies have shown that stretching the superficial neck and masticatory muscles reduces pain and muscle tension in TMJ dysfunction^58^, aligning with our findings. This effect may be due to the biomechanical interaction between the masticatory and neck muscles, where they function synergistically or antagonistically, supporting the cervical spine as flexors or extensors^5,58^. Furthermore, orofacial exercises improve muscle coordination, relax hypertonic muscles, increase ROM, and enhance muscle proprioception and endurance^59^. These findings suggest a synergistic mechanism between cervicoscapular and jaw exercises. The cervical and masticatory muscles are interconnected through anatomical and neural pathways, particularly via the trigeminocervical nucleus. Activation or relaxation of one region can influence the tone and coordination of the other. Cervicoscapular exercises, by improving neck and scapular alignment and reducing cervical muscle hyperactivity, may indirectly reduce the abnormal loading and compensatory overactivity of the masticatory muscles. Conversely, jaw exercises that target masticatory muscle relaxation and proprioception can contribute to better cervical stability by minimizing mandibular-induced forward head posture stress. This bidirectional relationship underlines the importance of comprehensive rehabilitation programs addressing both cervical and masticatory systems to optimize functional recovery.

The muscle stiffness results of this study showed significant decreases in both groups before and after the intervention. Greater anterior head positioning is associated with in-creased stiffness of the superficial neck muscles^60^. Exercise interventions targeting head posture have been shown to positively impact muscle stiffness. Postural exercises, often used for relieving neck and back pain, can also be applied to the orofacial region to alleviate muscle symptoms such as pain, tension, stiffness, and fatigue by improving head and mandibular alignment. These exercises include head posture correction, mandibular position correction, tongue postural exercises, and myofascial release^58^. Previous studies have also found that the passive muscle stiffness decreases immediately after static stretching^61^. Stretching aims to increase the muscle ligament flexibility, improve the range of motion or musculoskeletal capacity, and prevent injuries^56^. And the difference in the masseter muscle and temporal anterior muscle was greater in the experimental group than it was in the control group. In this study, we used the digastric facilitation posture exercise, which strengthens weakened infrahyoid muscles and stretches shortened suprahyoid muscles. The postural exercise included the correction of the mandibular position, tongue postural exercises, and myofascial release^50^, and has been proven to be effective in decreasing muscle stiffness^49^. The drop in stiffness after the exercise could be due to the relaxation of the tissues caused by the increased blood flow in and temperature of the treated tissue^49^. The temporalis and masseter muscles are part of the same neuromuscular chain (straight anterior chain) as the suprahyoid and infrahyoid muscles, responsible for the rolling movements of the head^62^. This is why these two muscles can be improved together through exercise.

In the intra-group comparison, both groups showed significant increases in the pressure pain threshold for the masseter and temporal anterior muscles. Previous studies have found that the pressure pain thresholds increase during and after stretching. Stretching has been shown to have a pain-inhibitory effect on skin pain perception^63^, stretching exercises enhance peripheral circulation, which facilitates overall relaxation and reduces muscle tension, leading to localized muscle relaxation. This process mitigates prolonged hypertonicity, particularly in muscles such as the masseter, thereby preventing the development of myofascial pain. Through these physiological adaptations, stretching and relaxation exercises contribute to soft tissue remodeling, reduce muscle stress, and ultimately result in an increased pressure pain threshold^64^, ultimately raising the PPT.

In the between-group comparison, the group performing the cervicoscapular complex exercises combined with the jaw exercises showed significant improvements in the variables directly related to temporomandibular joint function. In contrast, the control group demonstrated notable changes in variables associated with the cervical spine and scapulae. Despite both groups having the same total exercise duration, the experimental group showed a higher proportion of stable progress. Increased exercise engagement was associated with more noticeable improvements in head posture. The results also indicated that time influences the treatment effect, with the neck muscles having a more direct impact on neck posture, while the facial muscle exercises affected the entire head and neck musculature.

### Study limitations

However, the limitations of this study should also be acknowledged. First, it is difficult to control for the various daily activities and lifestyle factors that may influence the dependent variables, such as ergonomic conditions and physical activity levels. Additionally, the study was limited to university students with forward head posture, which may not fully represent other age groups or populations with different severity levels. Psychological factors, such as stress and anxiety, were also not considered, yet they may have influenced participants’ recovery and pain levels.

Based on these research limitations, it is recommended that future studies include a broader and more diverse participant pool, encompassing different age groups and individuals with varying degrees of forward head posture, to better assess the effectiveness of interventions across demographics. To control for lifestyle-related confounding factors, it is advised to utilize objective monitoring tools (e.g., wearable devices) for tracking daily activities and ergonomic conditions. Long-term follow-up assessments should also be conducted to evaluate the sustainability of intervention effects. Moreover, future studies should consider the influence of psychological factors, such as stress and anxiety, on recovery outcomes. For practical applications, it is suggested to integrate posture training into daily routines through mobile applications, educational programs, or workplace interventions. Finally, a multifaceted approach that combines posture training with ergonomic adjustments and stress management strategies may further enhance the effectiveness of interventions.

## Conclusion

This study demonstrated that both cervicoscapular exercises alone and the combination of jaw and cervicoscapular exercises significantly improved mouth opening range of motion (ROM), muscle mechanical properties, and pain threshold in individuals with cervical extension-type posture. Notably, the addition of jaw exercises led to greater improvements in protrusive excursion, masseter muscle tone, and muscle stiffness compared to cervicoscapular exercises alone. These findings underscore the clinical relevance of integrating jaw exercises into rehabilitation protocols for individuals with cervical extension-type posture and temporomandibular joint (TMJ) dysfunction. Rehabilitation professionals are therefore encouraged to incorporate combined jaw and cervicoscapular training approaches to optimize musculoskeletal function, reduce pain, and enhance therapeutic outcomes in this population.

## Data Availability

All data generated or analyzed during this study are included in the published article.
